# Transcriptome divergence between developmental senescence and premature senescence in *Nicotiana tabacum* L.

**DOI:** 10.1038/s41598-020-77395-2

**Published:** 2020-11-25

**Authors:** Zhe Zhao, Jia-Wen Zhang, Shao-Hao Lu, Hong Zhang, Fang Liu, Bo Fu, Ming-Qin Zhao, Hui Liu

**Affiliations:** College of Tobacco Science, Henan Agricultural University, Zhengzhou, 450002 People’s Republic of China

**Keywords:** Plant hormones, Plant physiology, Plant stress responses

## Abstract

Senescence is a degenerative process triggered by intricate and coordinated regulatory networks, and the mechanisms of age-dependent senescence and stress-induced premature senescence still remain largely elusive. Thus we selected leaf samples of developmental senescence (DS) and premature senescence (PS) to reveal the regulatory divergence. Senescent leaves were confirmed by yellowing symptom and physiological measurement. A total of 1171 and 309 genes (DEGs) were significantly expressed respectively in the whole process of DS and PS. Up-regulated DEGs in PS were mostly related to ion transport, while the down-regulated DEGs were mainly associated with oxidoreductase activity and sesquiterpenoid and triterpenoid biosynthesis. In DS, photosynthesis, precursor metabolites and energy, protein processing in endoplasmic reticulum, flavonoid biosynthesis were notable. Moreover, we found the vital pathways shared by DS and PS, of which the DEGs were analyzed further via protein–protein interaction (PPI) network analysis to explore the alteration responding to two types of senescence. In addition, plant hormone transduction pathway was mapped by related DEGs, suggesting that ABA and ethylene signaling played pivotal roles in formulating the distinction of DS and PS. Finally, we conducted a model containing oxidative stress and ABA signaling as two hub points, which highlighted the major difference and predicted the possible mechanism under DS and PS. This work gained new insight into molecular divergence of developmental senescence and premature senescence and would provide reference on potential mechanism initiating and motivating senescence for further study.

## Introduction

Senescence is the final phase of leaf development, which contribute to reproduction and survival^1^, as well as the recycling and reallocation of valuable nutrients^2^. As a disintegrated and degenerated process, senescence is concomitant with an intensive restructuring of cells, involving the breakdown of macromolecules, such as chlorophyll, proteins, nucleic acids and membrane lipids^3^, the remobilization of nutrients, the decline in photosynthesis^4,5^, which was triggered through the intricate regulatory networks of transcription factors^6,7^, hormones, reactive oxygen species (ROS) and so on^8^.

As signals regulating senescence, plant hormones played vital roles both in developmental senescence and stress-induced premature senescence^2^. The role of auxin, cytokinins (CKs), gibberellin (GA), abscisic acid (ABA), ethylene, jasmonic acid (JA) and so on, may function independently or with cross-talk in a complex and coordinated network^1^. For example, it was reported that jasmonate could interact with auxin, ethylene, and gibberellin signaling pathway to regulate leaf senescence^10^. Exogenous cytokinins, antagonizing the function of abscisic acid, could redistribute soluble sugars and counteract premature senescence^11^. PP2A regulatory subunit PP2A-B'γ, which participated in negatively controlling the expression of salicylic acid-related defense genes, had been proved to promote senescence^12^. ABA was regarded as a positive regulator of leaf senescence, and the exogenous application of which could accelerate chlorophyll degradation^13^. ABA receptor PYL9 had been proved to promote drought resistance and leaf senescence^14^.

Transcription factors (TFs), which switch on the manipulation of gene expression, have been widely reported as the regulators of leaf senescence^15^. For example, WRKY DNA-binding protein 45 (WRKY45) was reported as a positive regulator of age-triggered leaf senescence^16^. The expression of a NAC transcription factor-SlNAP2, increased during age-dependent and dark-induced leaf senescence^17^. Moreover, Guo^18^ demonstrated that the overexpression of WRKY75 would accelerate leaf senescence by promoting SA production and suppressing H_2_O_2_ scavenging according to the expression of SID2 and CAT2. The NAC transcription factor, SiNAC1, participated in a positive feedback loop via ABA biosynthesis and leaf senescence^19^. Additionally, MYB transcription factor, OsMYB102, involved in the regulation of leaf senescence, through the downregulated ABA biosynthesis and signaling response^20^.

Intrinsically, the initiation of senescence is the consequence of integrated signals, including endogenous and environmental signals^4^. The developmental senescence, which was a coordinated physiological process and being induced by the endogenous factors^21^, has been studied in a large variety of plants using high-throughput method, such as *Arabidopsis*^22^, wheat^23^, maize^24^, *Gossypium hirsutum* L.^25^, tobacco^26^, sorghum^27^, soybean leaves^28^, sunflower^29^, *Lonicera macranthoides* leaves^30^, grape berry^31^, pear^32^, and so on. On the other hand, when confronted with uncomfortable external factors, plants were inclined to start an ‘escape’ or protective strategy, to decrease canopy size and ensure the optimal survival for next generation^4,33^, which resulted in premature senescence. In recent studies, it has been proved that a wide variety of abiotic and biotic stresses, such as drought^34^, heat^35^, salt^36^, would trigger premature senescence^2^. Besides, in nature, it is more frequent for plants to suffer multiple simultaneous or sequential stress conditions than a single individual stress^37,38^. Therefore, the study on the mechanism of combined stresses-induced senescence in the field was a practical and promising work.

In this study, we chose tobacco leaves as materials to identify some major signal and pathway changes between premature and developmental senescence. Given that the questions of senescence on signal transduction and cell perception still remain unsolved^2^, the comparison of gene expression patterns between developmental senescence and premature senescence will lay a crucial foundation on further depicting the signal transduction and molecular regulation of senescence, and will help to deepen the understanding and provide a reference on enhancing the stress tolerance in plants.

## Results

### Morphologic and biochemical changes during developmental senescence and premature senescence

**Figure 1:**
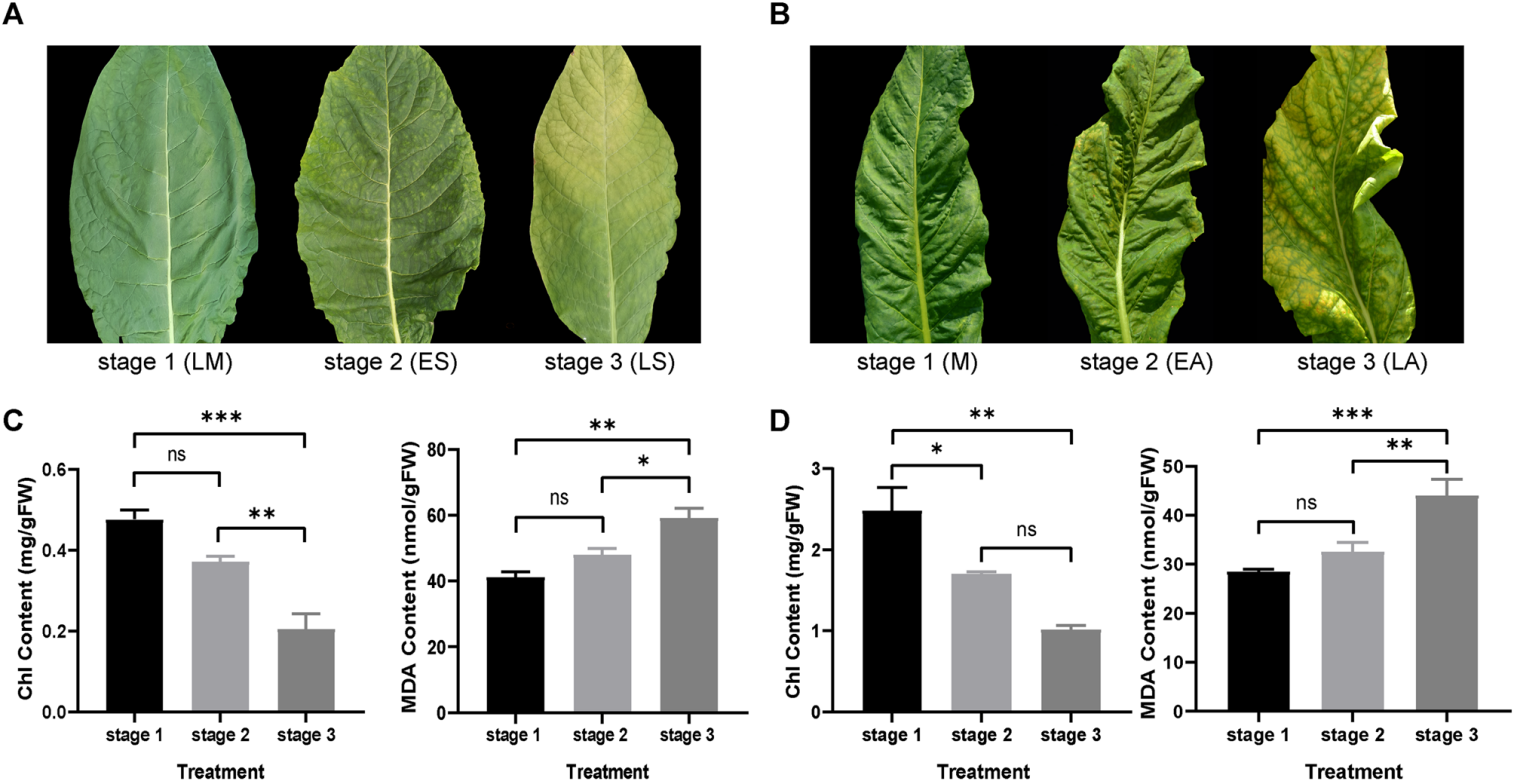
Morphologic characterization, chlorophyll (Chl) and malondialdehyde (MDA) contents in plants. (**A**) phenotype of developmental senescence (DS). (**B**) phenotype of premature senescence (PS). (**C**) Chl and MDA contents of leaves in DS. (**D**) Chl and MDA contents of leaves in PS. Results of Chl and MDA are shown as means ± SE. “ns” means no significant difference. *, ** and *** represent significant difference at p < 0.05, 0.01 and 0.001 respectively. **B** and **D** were adapted from our previous work^49^.

It has been reported that the visible leaf yellowing rate can be the evidence to confirm senescence^39,40^. In this study, we chose three stage points from maturity to late senescence. At stage 1, the leaves showed green, fully expanded and no signs of yellowing. Then there was about 10% leaf yellowing rate in leaf blade, which was regarded as stage 2. With senescence going on, almost half blade turned into yellow (50% yellowing rate), indicating the appearance of stage 3. In DS, we termed stage 1, stage 2 and stage 3 as LM, ES and LS, and in PS they were named as M, EA and LA (Fig. 1A,B). Both in DS and PS, Chl content decreased and MDA content increased significantly (Fig. 1C,D), indicating the transition from maturity to senescence of leaves.

### RNA-Seq analysis and DEGs identification

Totally 18 RNA libraries were sequenced from three stages of DS and PS. As shown in Supplementary Table S1, we have generated at least 49.32 Mb raw reads from each library, and 47.64–90.71 Mb clean reads were obtained after filtering out low quality tags, with more than 91.675% Q30 rate, which demonstrated high confidence of clean data. Then the sequences were mapped with *Nicotiana tabacum* genome^41^. As a result, 67.4–94.94% reads for samples were totally mapped to this genome.

Differential expression analysis was performed to detect DEGs of DS and PS. In DS, we detected 1292 DEGs between ES and LM and 18287 DEGs between LS and LM. Both the two stages shared 1171 common DEGs, out of which 646 genes were significantly up-regulated and 505 genes were down-regulated (supplementary Figure S1A). As for PS, 775 genes and 2559 genes were identified to be DEGs in EA and LA respectively compared with M. The total number of 309 genes (51 up- and 253 down-regulated genes) were found expressed both in early and late PS (supplementary Figure S1B), which should be put more focus on (supplementary table S2).

### GO analysis of significantly enriched terms and common terms respectively in up-and down-regulated DEGs

**Figure 2:**
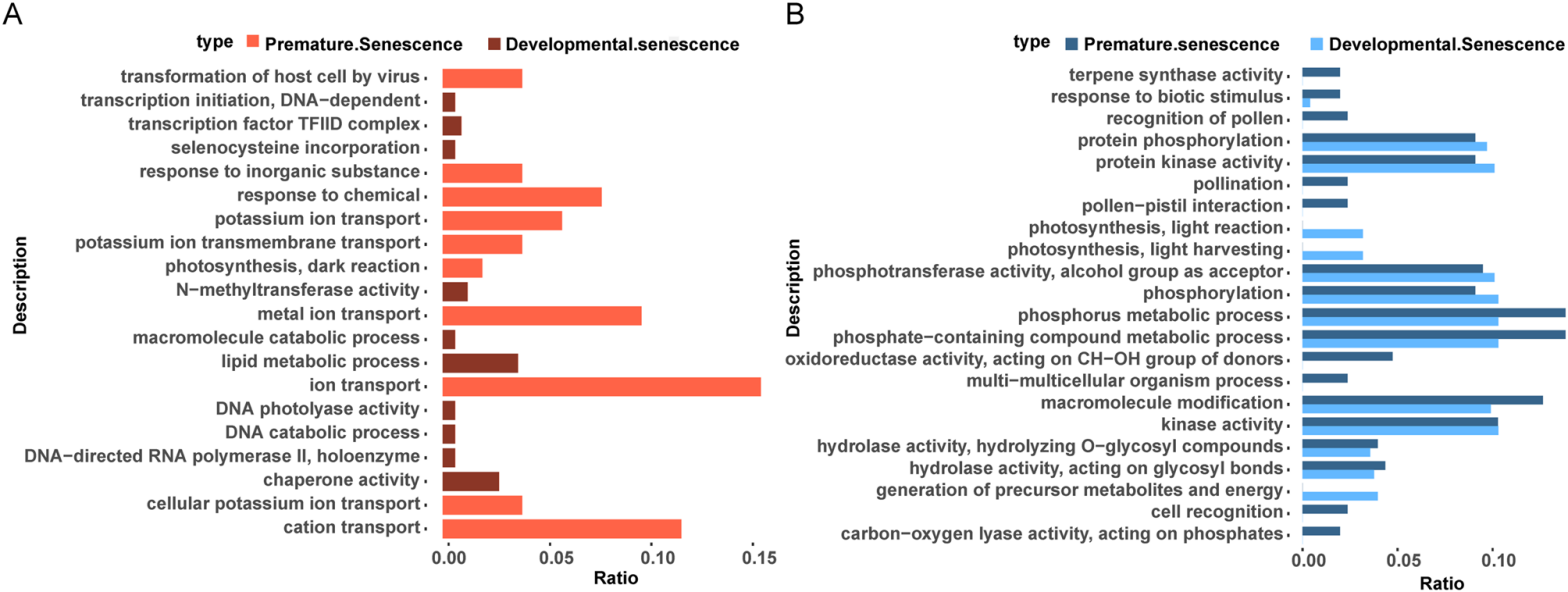
Top 10 significantly overrepresented GO terms and common GO terms in developmental and premature senescence. (**A**) Barplot of GO terms in up-regulated DEGs. (**B**) Barplot of GO terms in down-regulated DEGs. The x-axis represents the ratio of the number of enriched genes to the number of up- or down-regulated DEGs independently in developmental senescence and premature senescence. The y-axis shows the enriched GO terms.

To reveal the molecular difference between DS and PS, we mainly analyzed the common DEGs respectively from DS (1171 DEGs) and PS (309 DEGs), which were more relevant to senescence on account of their high expression in two senescent stages. The up- and down-regulated common DEGs of DS and PS were independently annotated to GO term. Top ten significantly enriched GO terms and the common GO terms for the two cultivars with the criteria of p-value < 0.05 were selected. Up-regulated genes of PS were all enriched in biological process, the most significantly enriched GO terms were summarized ion transport, including cation, metal ion, potassium ion and cellular potassium ion. The highest overrepresented GO term of DS was lipid metabolic process, followed by chaperone activity, *N*-methyltransferase activity, transcription factor TFIID complex and so on, which covered biological process, cellular function and molecular function (Fig. 2A).

Down-regulated DEGs of DS and PS were enriched in eleven same GO terms containing biological process and molecular function, including phosphate-containing compound metabolic process, macromolecule modification, kinase activity, phosphorylation, protein kinase activity, protein phosphorylation, hydrolase activity, response to biotic stimulus etc., which were mainly implicated in protein phosphorylation (Fig. 2B). The rest of down-regulated genes in developmental senescence were involved in photosynthesis and generation of precursor metabolites and energy. As for premature senescence, oxidoreductase and carbon–oxygen lyase activity, pollination and cell recognition were involved. All these gave a preliminary recognition of DEGs’ function between DS and PS.

### KEGG analysis of up- and down-regulated DEGs in developmental and premature senescence

**Figure 3:**
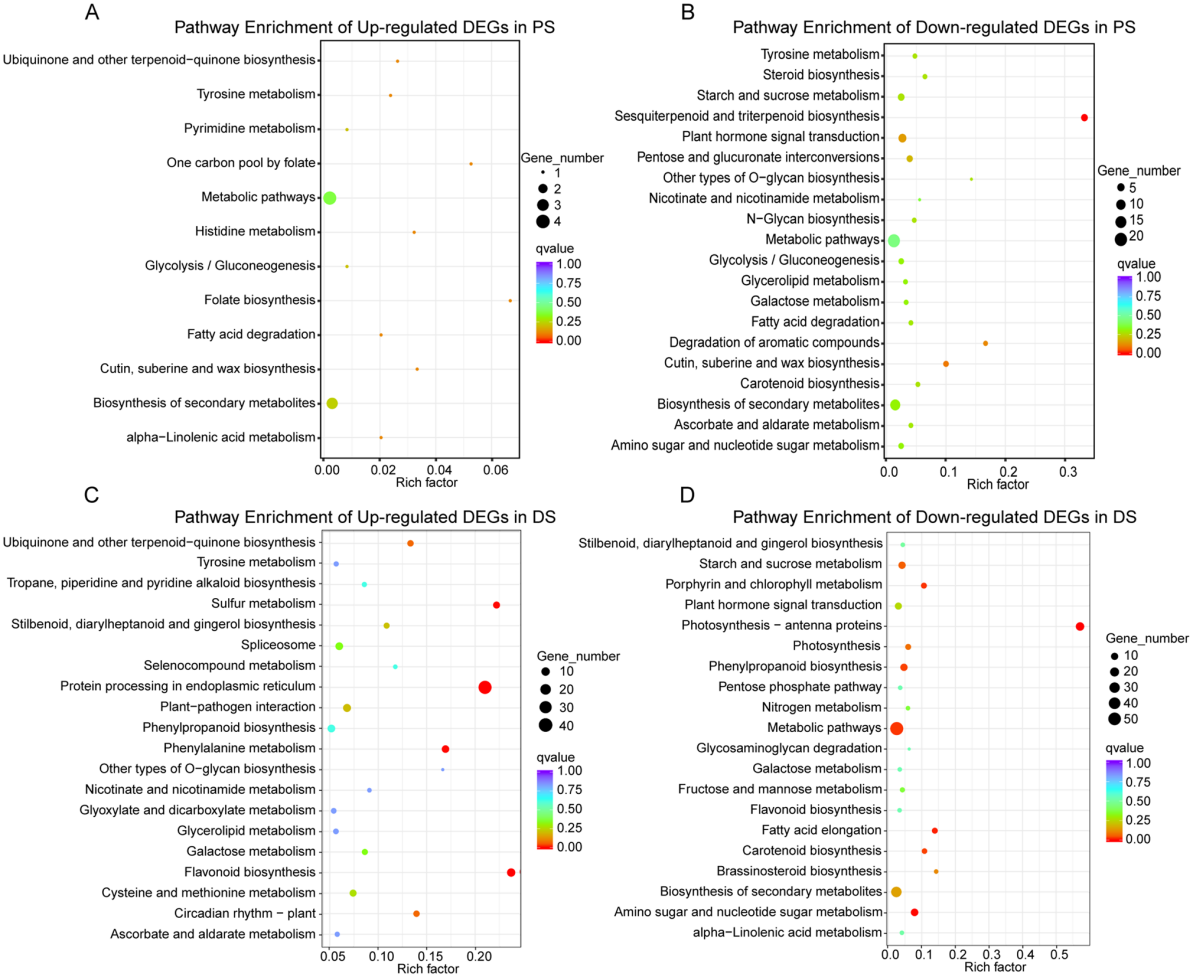
KEGG enrichment analysis of up- and down-regulated DEGs in PS and DS. PS, premature senescence. DS, developmental senescence. The x-axis means the ratio of numbers between enriched genes and background genes in this pathway. The y-axis represents KEGG pathway.

In the 309 and 1171 DEGs, up- and down-regulated DEGs were mapped to KEGG pathway separately using the KOBAS to further investigate the metabolic function (Fig. 3). In PS, notably, sesquiterpenoid and triterpenoid biosynthesis were significantly enriched (corrected p-value < 0.05), and the genes mapped to this pathway were down-regulated. There were much difference in significantly enriched metabolic pathways between PS and DS. As shown in Fig. 3C, four pathways, including protein processing in endoplasmic reticulum, flavonoid biosynthesis, sulfur metabolism and phenylalanine metabolism were significantly being mapped, which were up-regulated impressively. As for down-regulated DEGs in DS, most of them were mapped to the significant pathways, being consisted of photosynthesis—antenna proteins, amino sugar and nucleotide sugar metabolism, fatty acid elongation, porphyrin and chlorophyll metabolism, metabolic pathways, phenylpropanoid biosynthesis, and carotenoid biosynthesis (Supplementary Table S3).

### Common KEGG pathways shared by PS and DS

Based on the KEGG analysis of PS and DS, some pathways were shared by both two types of senescence. In order to further understand the pathways and key DEGs related to senescence, up- and down-regulated DEGs from two types of senescence were analyzed independently, and the common pathways had been listed in Tables 1 and 2. Regardless of metabolic pathways and biosynthesis of secondary metabolites, which participated in the whole process of senescence, glycolysis/gluconeogenesis, fatty acid degradation, ubiquinone and other terpenoid-quinone biosynthesis, pyrimidine, tyrosine and alpha-linolenic acid metabolism were involved in promoting senescence. For inhibiting the progress of senescence, galactose, starch and sucrose, amino sugar and nucleotide sugar, glycerolipid, glycerophospholipid and nitrogen metabolism, phenylpropanoid biosynthesis, base excision repair, plant hormone signal transduction, and protein processing in endoplasmic reticulum might function partially.

**Table 1:** Common pathways shared by PS and DS in KEGG enrichment analysis of up-regulated DEGs.

Up-regulated common KEGG pathways	p-values in PS	p-values in DS
Glycolysis/gluconeogenesis (sly00010)	0.176297182	0.446052315
Fatty acid degradation (sly00071)	0.075565339	0.826389427
Ubiquinone and other terpenoid-quinone biosynthesis (sly00130)	0.059417279	0.00586999
Pyrimidine metabolism (sly00240)	0.176297182	0.909915556
Tyrosine metabolism (sly00350)	0.065319774	0.272625816
Alpha-linolenic acid metabolism (sly00592)	0.075565339	0.80826022
Metabolic pathways (sly01100)	0.371454896	0.93252252
Biosynthesis of secondary metabolites (sly01110)	0.218476155	0.458746698

**Table 2:** Common pathways shared by PS and DS in KEGG enrichment analysis of down-regulated DEGs.

Down-regulated common KEGG pathways	p-values in PS	p-values in DS
Galactose metabolism (sly00052)	0.13969744	0.233559001
Starch and sucrose metabolism (sly00500)	0.067079335	0.005513379
Amino sugar and nucleotide sugar metabolism (sly00520)	0.143881706	1.97028E-05
Glycerolipid metabolism (sly00561)	0.146862018	0.671377151
Glycerophospholipid metabolism (sly00564)	0.26408271	0.780506097
Nitrogen metabolism (sly00910)	0.314382352	0.104525607
Phenylpropanoid biosynthesis (sly00940)	0.866936583	0.00224991
Metabolic pathways (sly01100)	0.240436906	0.001501138
Biosynthesis of secondary metabolites (sly01110)	0.12955448	0.077527221
Base excision repair (sly03410)	0.362990483	0.492997761
Plant hormone signal transduction (sly04075)	0.015292453	0.045463739
Protein processing in endoplasmic reticulum (sly04141)	0.460732297	0.878201245

### Recognition and analysis of key DEGs by PPI analysis

**Figure 4:**
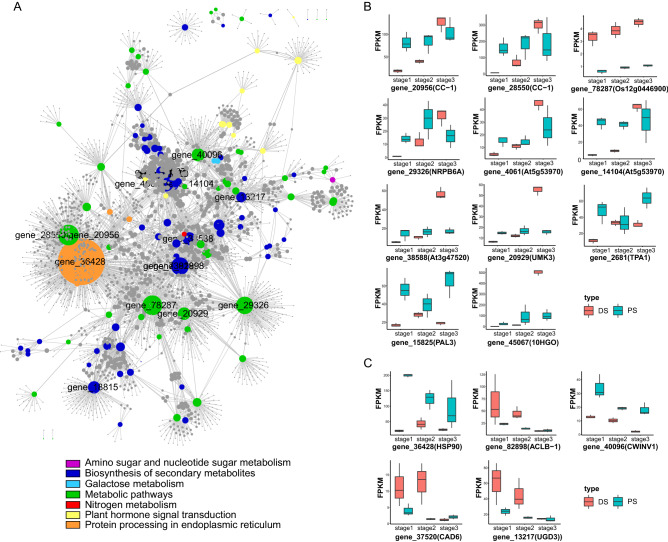
Identification and expression patterns of key regulatory genes in the common pathways. (**A**) Protein–protein interaction network of DEGs in common pathways. The size of each node indicates the interaction degree to other nodes. Nodes with gene ID represent genes whose degree was higher than 100. (**B**) Expression profiles of up-regulated genes with more than 100 degrees. (**C**) Expression profiles of down-regulated genes with more than 100 degrees.

To identify the contribution of gene expression patterns to the difference of senescence, we selected all the genes from the important up and down common pathways, with matched protein ID after being blast and the information of interaction, to make protein–protein network analysis (ppi analysis). The degree of interaction among genes and the number of connections from one to others were termed as “degree”. As shown in Fig. 4A, 17 genes, which were equipped with high degrees over 100, laid the most important foundation in the regulation network. Thus, the expression patterns of these genes could be analyzed further to seek for the specific and detailed difference between DS and PS.

Within common up-regulated pathways we identified 11 DEGs related to the encoding of CC-1, Os12g0446900, NRPB6A, At5g53970, At3g47520, UMK3, TPA1, PAL3 and 10HGO. Among these genes, some showed similar up-regulated trend both in DS and PS. However, other genes expressed different patterns, such as gene_29326, gene_4061, gene_14104, gene_38588, gene_20929 and so on, which need to be discussed further (Fig. 4B). As for the DEGs involved in down-regulated pathways (Fig. 4C), gene_82898, gene_40096 and gene_13217 were all down-regulated with the process of DS and PS, while gene_36428, named as *HSP90*, was down-regulated significantly in PS but irregularly expressed in DS. In addition, the expression of gene_37520 was irregular.

### Pathway analysis of plant hormone signal transduction related DEGs

**Figure 5:**
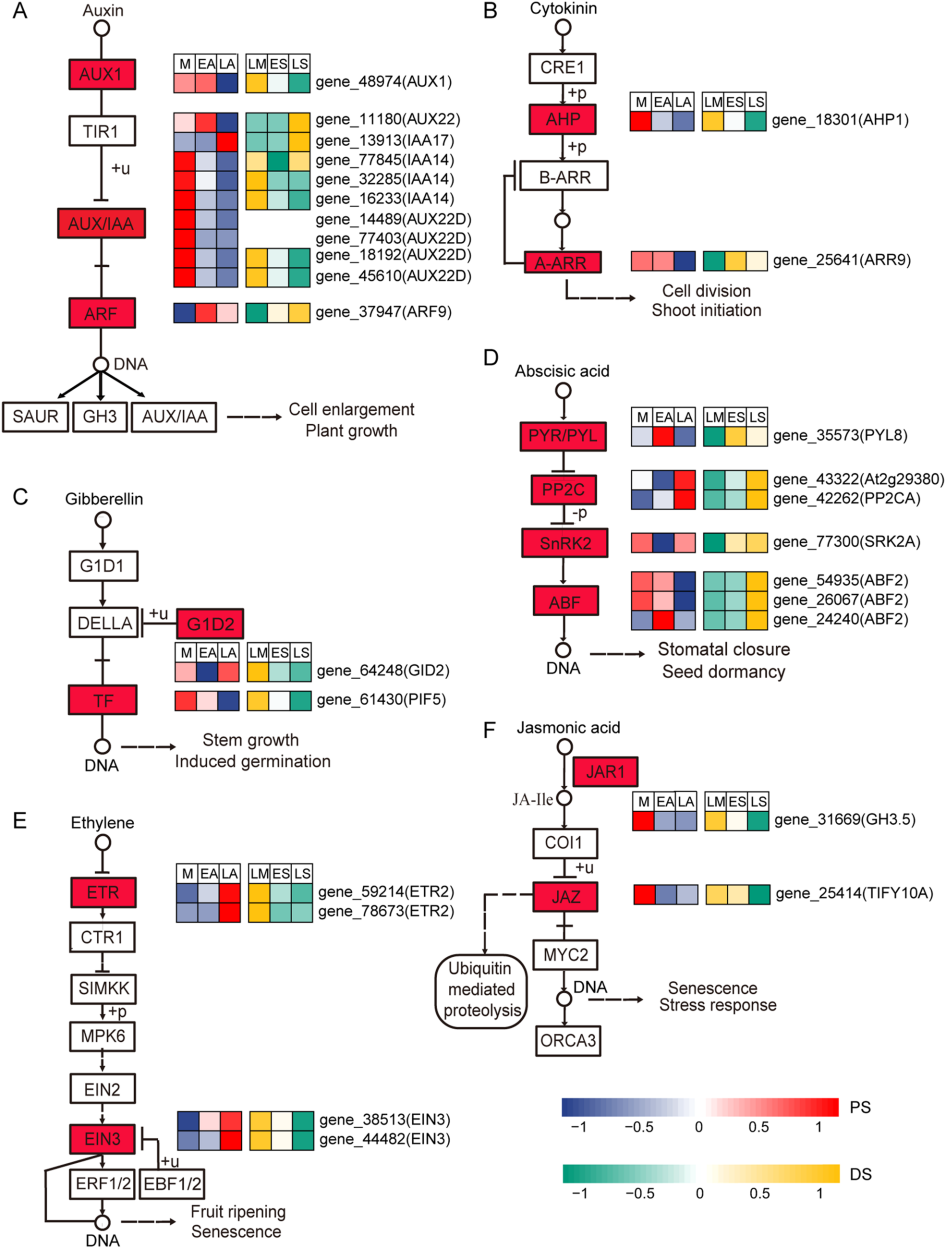
Expression patterns of DEGs enriched in hormone signal transduction pathway. (**A**) Auxin signal transduction pathway. (**B**) Cytokinin signal transduction pathway. (**C**) Gibberellin signal transduction pathway. (**D**) Abscisic acid signal transduction pathway. (**E**) Ethylene signal transduction pathway. (**F**) Jasmonic acid signal transduction pathway. Boxes means proteins or genes. Circles represent chemical compounds. Arrows indicate interactions and T-bars indicate inhibitory effects. Heatmaps of DEGs in DS and PS are performed respectively, and DEGs were located near the proteins they encoded, which have been shown in red boxes. KEGG pathway database^45^ was the reference of the pathways.

To investigate the function of plant hormone, 28 genes enriched in plant hormone signal transduction were examined, among which 8 genes belonged to the hormone signaling of PS, and the rest 20 genes were divided into the hormone regulation of DS. The expression profiles of these genes in two cultivars, implicated in auxin, cytokinin (CK), gibberellin (GA), abscisic acid (ABA), ethylene, and jasmonic acid transduction pathways, were shown in Fig. 5.

In auxin signal transduction pathway, the protein AUX1, AUX/IAA, ARF were expressed predominantly. Most genes named *AUX22D*, *IAA14*, and *AUX1* were down-regulated similarly in DS and PS, indicating the down-regulation of AUX1 and AUX/IAA in senescence. What to be noted was that gene_37947, encoding ARF, was up-regulated significantly in DS but down-regulated slightly in the last phase of PS (Fig. 5A). Two genes were enriched in cytokinin signal pathway (Fig. 5B), of which gene_25641 showed different expression patterns in two types of senescence and should be discussed further. Gibberellin signals cell growth, germination, and inhibits the process of senescence^42,43^. In this study, the differentially expressed genes implicated in GA signal transduction were down-regulated, except for the up-regulation of gene_64248 through EA to LA transition.

As the response to environmental stress and leaf senescence^44^, abscisic acid signal transduction pathway involved four differentially regulated proteins and 7 DEGs under study (Fig. 5D). The large proportion of these genes were up-regulated during developmental senescence, while the minority of them showed the downregulation, especially in premature senescence.

Intriguingly, it should be notable that the expression patterns of ETR and EIN3 were extremely opposite between developmental senescence and premature senescence (Fig. 5E), which may take part in modifying the onset of senescence. In addition, JAR1 and JAZ exposed similar modulation for whatever developmental or premature senescence, with the same gene expression patterns.

### Transcription factor analysis

Transcription factors (TFs) are vital proteins to modulate plant development and senescence. As we observed, among the 1171 DEGs and 309 DEGs, there were 526 and 33 TF transcripts in DS and PS separately (Supplementary table S4). The top 5 largest TF families were bHLH (53), MYB_related (44), B3 (31), bZIP (30) and NAC (27) families, with over 35% percentage of TF transcripts in DS. As for PS, MYB (7), HB (4), C2H2 (3), WRKY(3) and bHLH (2) families occupied the top 5 significance of TF families, which may participate in the regulation of premature senescence rather than the normal developmental senescence.

### Validation of the RNA-seq expression patterns by qRT-PCR

**Figure 6:**
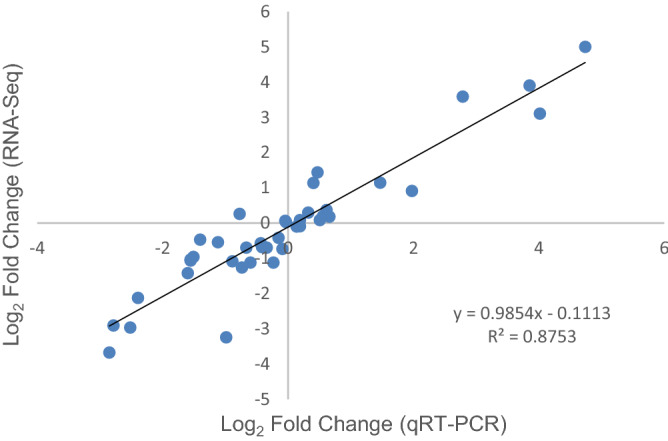
Correlation of expression patterns of selected genes from RNA-Seq and qRT-PCR.

To validate the reliability of the sequencing data, qRT-PCR analysis was performed. Under study ten differentially expressed genes were selected randomly to detect their expression profiles in three stages of DS and PS, respectively. Gene_22548(UNE10) and gene_58303(SPL5) belonged to the significantly expressed transcription factor, gene_38513(EIN3) and gene_26067(ABF2) were enriched in ethylene and ABA signal transduction pathway, gene_14104(At5g53970), gene_20929(UMK3), gene_36428(HSP90) and gene_82898(ACLB-1) were the key DEGs due to PPI analysis. Gene_34964(SAG12) and gene_35004(RBCS) were regarded as the senescence-associated genes^46,47^. Most of the transcript levels detected by qRT-PCR showed the similar patterns with the gene expression levels obtained from RNA-Seq (Supplementary Table S5, Supplementary Figure S2), and the accordance of transcriptome sequencing and qRT-PCR results were confirmed by a high correlation coefficient (R^2^) of 0.8753 (Fig. 6), which indicated the credibility of transcriptional data.

## Discussion

Senescence is the final phase of leaf period, which is the response of an integration of various exogenous signals and leaf age, involving systemic and intricate regulatory pathways^9^. Until now, though impressive progress has been made on senescence in some aspects, given its complicacy, there’s much unsettled on mechanism between developmental senescence and stress-induced premature senescence. In this study, we choose two types of senescence in tobacco plant, to discuss the difference among enriched pathways, function of important genes, signal transductions, to decipher the key factors regulating kinds of senescence and try to explore inner relationship in the coordinated strategy. The leaves of DS were selected according to the growth period and the phenotype of leaf yellowing rate first, then were confirmed by the rising MDA and decreased Chl contents, which represent the occurrence of senescence^48^. Leaves of PS, which were confirmed by meteorological factors, growing time, phenotype, physiological indicators and cell ultrastructure^49^, were used to analyze further, and the original transcriptome dataset of PS was the same as that of our previous work^49^.

When plants reached senescence, up-regulated DEGs from DS and PS belonged to different GO categories (Fig. 2A). Obviously, the GO term of ion transport accounted for the largest proportion in premature senescence, followed by the cation, metal and potassium ion transport, which implied that the function of up-regulated genes from PS mainly related to K+ and other ions’ transport and represented the possibility of ion transport implicated in early senescence. As reported, potassium served as the largest fraction of the inorganic osmotica, and its concentration changed the closed and open states of stomata from guard cells^50,51^, the aperture of which depends on the osmotic solute accumulation and loss^52^. Moreover, interaction between ABA synthesis and stomatal closure triggers water loss and leaf senescence^53^, which alters ion transport activity^54^. In DS, most of up-regulated DEGs were enriched in lipid metabolic process, which has been found low-expressed in aged senescent sorghum, up-regulated in dark-induced sorghum senescence^27^ and similarly enriched in lipid metabolic process in apricot under drought stress^55^, deducing that the pathway may be involved in most senescent process.

Phosphorylation process, served as a regulatory device in plant growth, can affect enzyme activities in direct ways^56^. In the analysis of down-regulated genes during senescence (Fig. 2B), DS and PS both shared the GO terms mainly associated with protein phosphorylation and protein kinase activity, the two of which exhibited tight connection in function. Phosphorylation aims at rapid regulation of protein function, such as ion channel activities in guard cells, and phosphorylation exits extensively in guard cell signaling^57^. Another macromolecule modification occupied a higher percentage of the common down-regulated GO terms, which had been found in tea plants under drought, heat and their combined stresses^58^.

In addition, except for the common terms, most genes of PS were correlative to the oxidoreductase activity, which belonged to antioxidant system, demonstrating the resistance to stresses. As a natural process, it was conceivable that the function of down-regulated genes in DS focused on photosynthesis and generation of precursor metabolites and energy, to fulfill the accumulation and reallocation of valuable resources.

The results of KEGG enrichment of PS exhibited the mere significantly overrepresented pathway-sesquiterpenoid and triterpenoid biosynthesis (Fig. 3B). Referring to previous study, sesquiterpenoid and triterpenoid can be induced increasingly by exogenous methyl jasmonate^59^. In this study, given the down-regulated expression of *JAR1* and *JAZ* (Fig. 5F), which indirectly concluded the low content of JA, the sesquiterpenoid and triterpenoid biosynthesis showed negative patterns during PS, being coincident with the previous result^57^. Likewise, in senescing callus tissue of *Aquilaria malaccensis*, the number of genes and enzymes in sesquiterpenoid and triterpenoid biosynthesis were lower than healthy callus tissue^60^.

Previous findings indicated that the endoplasmic reticulum was served as initiator of programed cell death in plants^61^. In accordance with the findings, genes of protein processing in endoplasmic reticulum pathway were up-regulated during DS (Fig. 3C). Flavonoid biosynthesis pathway was significantly enriched in DS, which was regarded as potent antioxidant and possessed the ability of inhibiting auto-oxidation and scavenging free radicals^62,63^, suggesting the suppression of ROS significantly depend on flavonoid in developmental senescence. this flavonoid biosynthesis, as reported, was overrepresented in senescent sorghum leaves likewise^27^. Furthermore, the up-regulated phenylalanine metabolism pathway, which was overrepresented in DS, had been found existed in naturally senescent sorghum and maize leaves^27,64^.

Antenna complexes, which were binded by Chl*b* and antenna proteins, affect light harvesting and efficiency of photosynthesis. In some researches, contents of part antenna proteins, as well as ABA signaling, were influenced by the levels of Chl*b*^65,66^. Therefore, the two pathways, photosynthesis-antenna proteins and porphyrin and chlorophyll metabolism, were both significantly down-regulated in DS (Fig. 3D), and the results was consistent with the findings in cotton during leaf senescence^25^. Additionally, carotenoid biosynthesis, which could protect photosynthetic apparatus from ROS attacking^67^ and provided precursors of ABA^68,69^, were predictably down-regulated in DS.

Except for the individual significantly enriched pathways, DS and PS shared common enriched pathways (Tables 1, 2), with the gene expression patterns in these pathways differing a lot. Given the protein interaction for each gene, we selected 17 genes which possessed more connections with other proteins and mean the higher importance in shared pathways (Fig. 4). gene_4061 and gene_14104(At5g53970), known as *TAT2*, encoding tyrosine aminotransferase which is strongly induced upon aging and coronatine treatment^70^, were up-regulated in DS but showed no significant changes in PS. In LSD 3.0^71^, *At5g53970* was sensed as a senescence-associated gene and took part in Arabidopsis senescence^15^. According to their expression profiles, it was predicted that the gene *At5g53970* could not significantly execute all types of cell death. *At3g47520*, named as *MDH*, encodes a protein with NAD-dependent malate dehydrogenase activity, which was probably essential for early chloroplast development^72^ and involved in the inactivation of redox regulator of catalase (CAT) via malate oxidation^73^. It was suggested that in the process of DS the activity of CAT was suppressed due to the increasing expression of gene_38588(At3g47520). UMP-CMP kinases produced pyrimidines and increased in antioxidant levels^74^, however, in this study the gene UMP-CMP kinase 3(*UMK3*) was significantly up-regulated in developmental senescence but with no significant difference under stress. *TPA1* and *PAL3* possessed similar expression profiles independently, as two genes encoding phenylalanine ammonia-lyase, which was the first enzyme of phenylpropanoid pathway^75^. Given that the activation of phenylpropanoid pathway offered a source of nonenzymatic antioxidants responding to oxidative stress^76^, there was a putatively preliminary observation that DS suffered oxidative stress in the early stage of senescence but PS suffered in late stage (Fig. 4B).

When inactivating or silencing the gene *HSP90*, the results may lead to the disturbance of H_2_O_2_ balance^77^, accumulation of damaged ubiquitinated proteins and cell death^78^. Under study, the down-regulated gene_36428(HSP90) exhibited a predominant decline in PS (Fig. 4B), and it took over the most pivotal role in monitoring all the pathways for its highest degree in PPI analysis (Fig. 4A), demonstrating the response of premature senescence was quite possibly related to the inhibition of *HSP90*. *CWINV1* encoding cell-wall invertases were both down-regulated in DS and PS, which modulated plant metabolism during defence responses^79^, showing the similarity between DS and PS.

Plant hormones, served as predominant signaling components, regulate not only age-dependent but also stress-induced leaf senescence^2^. Most of the genes implicated in abscisic acid (ABA), ethylene (ET) and jasmonic acid (JA) function positively in senescence signaling pathways, whereas auxins, cytokinins and gibberellins mainly delay the process of senescence^25,80^. Genes encoding AUX1 and AUX/IAA were mostly down-regulated, with gene_11180(AUX22) and gene_13913(IAA17) playing the opposite roles compared with others (Fig. 5A). *IAA14*, described as indole-3-acetic acid inducible 14, were widely studied functioning as a negative regulator of *ARF7/19*^81,82^. In this data, *IAA14* owned the roughly contrary expression pattern compared with *ARF9* and *IAA17*, deducing its possible role in negatively regulating ARF9 and motivating the converse function of *IAA17*.

Cytokinins are chemical signals that control plant developmental processes and environmental responses^83^. In cytokinin signal transduction pathway, the same low expressions of gene_18301 in two cultivars led to the down-regulation of AHP and B-ARR, consistent with the function of the up-regulation of gene_25641 in DS, which encoded A-ARR and inhibited the expression of B-ARR. However, the expression of gene_25641 played a quite opposite role in PS compared with DS (Fig. 5B). Gene *ARR9* can be repressed by oxidative stress via the transcription factor *CRF6*, which was related to the inhibition of dark-induced senescence^84^. Thus, it was understood that in PS the gene ARR9 were repressed under oxidative stress, reflecting the different oxidative levels in the two processes of senescence.

In previous studies, ABA could promote senescence by various approaches, such as causing ethylene biosynthesis^85^, activating sucrose nonfermenting 1-related protein kinase 2s (SnRK2s)^14^, interacting with the stomatal closure during stress^86^, and mediating ABA-triggered Chl degradation via regulating *ABF2*^87^. Referring to the expression patterns of genes enriched in ABA signal transduction pathway, the most impressive distinction belonged to *ABF2*, highlighting its notable down regulation in PS (Fig. 5D). *ABF2* was reported to take part in adaptive processes to abiotic stresses and occupy an active role in stomatal closure^88^. Thus, it can be deduced that the premature senescence process may be associated with ABF and stoma in guard cell, while age-dependent senescence was correlative to ABF and Chl degradation.

Ethylene play an important role in modulating senescence^89^. *ETR2*, not only served as ethylene receptor, but also affected ABA sensitivity by indirectly affecting the expression of genes encoding ABA signaling proteins, including *ABF2* and so on^90^. Moreover, cross talk between ethylene and other hormone signal transduction pathways involved the EIN3 signal^91^, which were expressed consistently with *ETR2* in our study (Fig. 5E), intensively suggesting the under interaction among *ETR2*, *EIN3* and *ABF2*.

**Figure 7:**
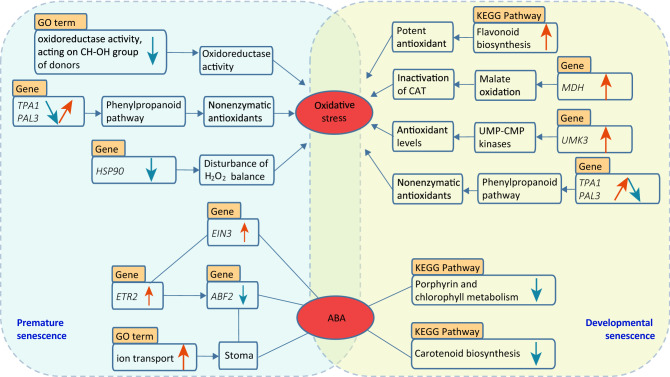
Model of regulatory divergence between DS and PS. The orange and blue arrows in the box represent up and down regulation respectively. Arrows and lines connected boxes indicate the interaction.

Thus we put forward a model aimed at pointing major divergence between premature and developmental senescence (Fig. 7). Referring to the analysis above, the oxidative stress and ABA signaling was extracted as two hub points to decipher the difference.

As shown in Fig. 7, The first concern correlative to oxidative stress in PS belonged to the GO term of oxidoreductase activity. This term was down-regulated in PS but not enriched in DS, representing its functioning in the process of PS. The expression patterns of *TPA1* and *PAL3* in PS, which encoded the first enzyme of phenylpropanoid pathway and offered a source of nonenzymatic antioxidants^75,76^, should also be noticed as the response of oxidative stress. Another attention for oxidative stress we should pay on was the expression patterns of *HSP90*, which was involved in the disturbance of H_2_O_2_^77^. As for oxidative stress in DS, flavonoid biosynthesis was significantly up-regulated in DS, but not found enriched in PS. Gene *MDH*, involved in the inactivation of catalase (CAT) via malate oxidation^73^, was up-regulated in DS. UMP-CMP kinases increased in antioxidant levels^74^, however, in this study the gene *UMK3* was significantly up-regulated in developmental senescence but no significant difference under stress.

The other hub point, ABA, was considered as a regulator promoting senescence. In this study, ABA was related to hormones signals, stoma closure, chlorophyll metabolism and carotenoid biosynthesis. *EIN3* possessed the same expression patterns with *ETR2*, and it worked for the crosstalk between ethylene and other hormone signal transduction pathway^91^. Moreover, the interaction between *ETR2* and *ABF2* also affected ABA sensitivity. Meanwhile, *ABF2* and the up-regulated GO terms of ion transport played roles in stoma closure^53,88^. In DS, the down-regulated two pathways, which influenced ABA signaling^65,66^ and offered precursors of ABA^68,69^, were also associated with ABA.

Taken together, this work drew a holistic and detailed picture for age-dependent senescence and combined-stresses induced premature senescence in *Nicotiana tabacum*, provided the potential mechanism initiating and motivating senescence when plants facing natural aging and stresses, and gave reference to the under interaction of genes and pathways for further study to conquer the high hill on signaling and execution of senescence.

## Materials and methods

### Plant materials and leaf sampling

Two *Nicotiana tabacum* cultivars Yunyan 87 (Y87) and Shiyan Number 1 (SN1) were used to study premature senescence (PS) and developmental senescence (DS) respectively. SN1 was cultivated at the experiment station in Shifang, Sichuan province, China (lat 31°38′N, long 104°09′E). After being sterilized with 2% sodium hypochlorite for 10 min, seeds sprouted in plastic pots. When the fifth true leaves occurred, seedlings were transplanted into soil on May 4, 2018. The soil of the field was paddy soil that contained 3.01 g/kg organic matter and 120.0, 38.1 and 89 mg kg^−1^ available N, P and K respectively. The fifteenth leaves of SN1, counted from the bottom of the plant, were collected on August 5th, August 9th and August 15. The growth period of SN1 was regarded as developmental senescence, following the typical patterns of leaf colors changing from green to yellow^27^.

Seedlings of Y87 were grown at an experimental field in Guangchang, Jiangxi province, China (Lat 26°33′N, Long 116°53′E). The adverse meteorological conditions and early senescence phenotypes of part plants had been observed continuously within past 3 years (Supplementary Table S6). Soil was turned over deeply and fertilized equally. On June 23rd, 2017, when plenty of upper leaves were mature but not senescent and others were senescent, we took the samples from maturity to late senescence, divided them into three stages and attributed the growth difference as premature senescence, which were proved by leaf yellowing rate, physiological indicators and cell ultrastructure^49^.

All samples of SN1 and Y87 were collected from the middle part of the fifteenth leaves’ blade, and frozen in liquid nitrogen and stored at − 80 ℃. Three biological replicates per stage were chosen for RNA-Seq and physiological analysis, and each biological replicate was pooled from three plants which were selected randomly to avoid potential effects of nutrition and position.

### Chlorophyll and MDA content quantification

Three replicated samples for each senescence stage were used to perform physiological analysis. As described by the method of Liu^92^, the measurement of chlorophyll (Chl) followed the procedures of extraction, storage, centrifugation and colorimetry. The absorbance of supernatant was measured using a spectrophotometer (UV-1780, Shimadzu, Japan). Malondialdehyde (MDA) content was quantified by the instruction of Saher^93^.

### RNA preparation and Illumina sequencing

Total RNA was extracted from frozen tissue using Spectrum Plant Total RNA Kit (Sigma-Aldrich, St. Louis, USA). RNA purity, concentration and integrity were assessed using NanoPhotometer spectrophotometer (IMPLEN, CA, USA), Qubit RNA Assay Kit in Qubit 2.0 Flurometer (Life Technologies, CA, USA) and RNA Nano 6000 Assay Kit of the Agilent Bioanalyzer 2100 system respectively. Sequencing libraries were generated using NEBNext Ultra RNA Library Prep Kit for Illumina (NEB, USA) according to manufacturer’s instructions. Then the library preparations were sequenced on an Illumina Hiseq 4000 platform and paired-end 125/150 bp reads were generated. Eighteen libraries including three biological replicates per treatment were constructed. Among these libraries, nine sequencing libraries of PS were employed from our previous study^49^. After removing low quality reads and reads containing adapter and poly-N, two cultivars’ clean reads were both mapped to the tobacco reference genome (ftp://anonymous@ftp.solgenomics.net/genomes/Nicotiana_tabacum/assembly/Ntab-K326_AWOJ-SS.fa.gz).

### RNA-Seq data analysis

Gene function was annotated based on the following databases: Nr (NCBI non-redundant protein sequences), Swiss-Prot (A manually annotated and reviewed protein sequence database), KO (KEGG Ortholog database) and GO (Gene Ontology). Quantification of gene expression levels were estimated by fragments per kilobase of transcript per million fragments mapped (FPKM). DESeq R package (version 1.10.1) (https://www.bioconductor.org/packages/release/bioc/html/DESeq.html) was used to conduct differential expression analysis, and Benjamini and Hochberg’s approach was performed to adjust the resulting p values to control the false discovery rate. In this study, genes with fold change ≥ 1 in Y87 and SN1, as well as p-values ≤ 0.05 (Y87) and adjusted p-values ≤ 0.05 (SN1), were identified as differentially expressed genes (DEGs). GO enrichment analysis of the DEGs was implemented by the GOseq R packages^94^ (v 1.40.0) (https://www.bioconductor.org/packages/release/bioc/html/goseq.html) based on Wallenius non-central hyper-geometric distribution. We used KEGG Orthology Based Annotation System (KOBAS)^95^ software (v 2.0) (https://kobas.cbi.pku.edu.cn/kobas3/?t=1) to test the statistical enrichment of DEGs in KEGG pathways, and Cytoscape^96^ (v 3.7.2) (https://cytoscape.org) to visualize the Protein–Protein Interaction (PPI) among genes, which were blast to the genome of *Solanum* *lycopersicum* and obtain the predicted PPI in the STRING database (https://string-db.org/).

### Real-time quantitative RT-PCR (qRT-PCR)

The extracted total RNA samples for RNA-Seq were reused to perform qRT-PCR test. Expressions of eight genes in three stages were measured via qRT-PCR. Based on the mRNA sequences obtained from the NCBI, the primer sequences (Supplementary Table S7) were designed in the laboratory and synthesized by Generay Biotech (Generay, PRC). The yield of RNA was determined using a NanoDrop 2000 spectrophotometer (Thermo Scientific, USA), and the integrity was evaluated through agarose gel electrophoresis stained with ethidium bromide. Reverse transcription (RT) reaction and real-time PCR were performed as previously described^48^. The expression levels of mRNAs were normalized by the internal control gene *L25*, and were calculated using the 2^−ΔΔCt^ method^97^.

## Supplementary information

Supplementary Figure S1.

Supplementary Figure S2.

Supplementary Table S1.

Supplementary Table S2.

Supplementary Table S3.

Supplementary Table S4.

Supplementary Table S5.

Supplementary Table S6.

Supplementary Table S7.

Supplementary Captions.

## Data Availability

The raw reads of RNA-Seq were deposited into NCBI SRA with BioProject accession number PRJNA672258.
