# Supplementary figures and images for: Transcriptome divergence between developmental senescence and premature senescence in Nicotiana tabacum L
Source: Sci Rep. 2020 Nov 25;10:20556. doi: 10.1038/s41598-020-77395-2 (PMC7688636; doi:10.1038/s41598-020-77395-2)

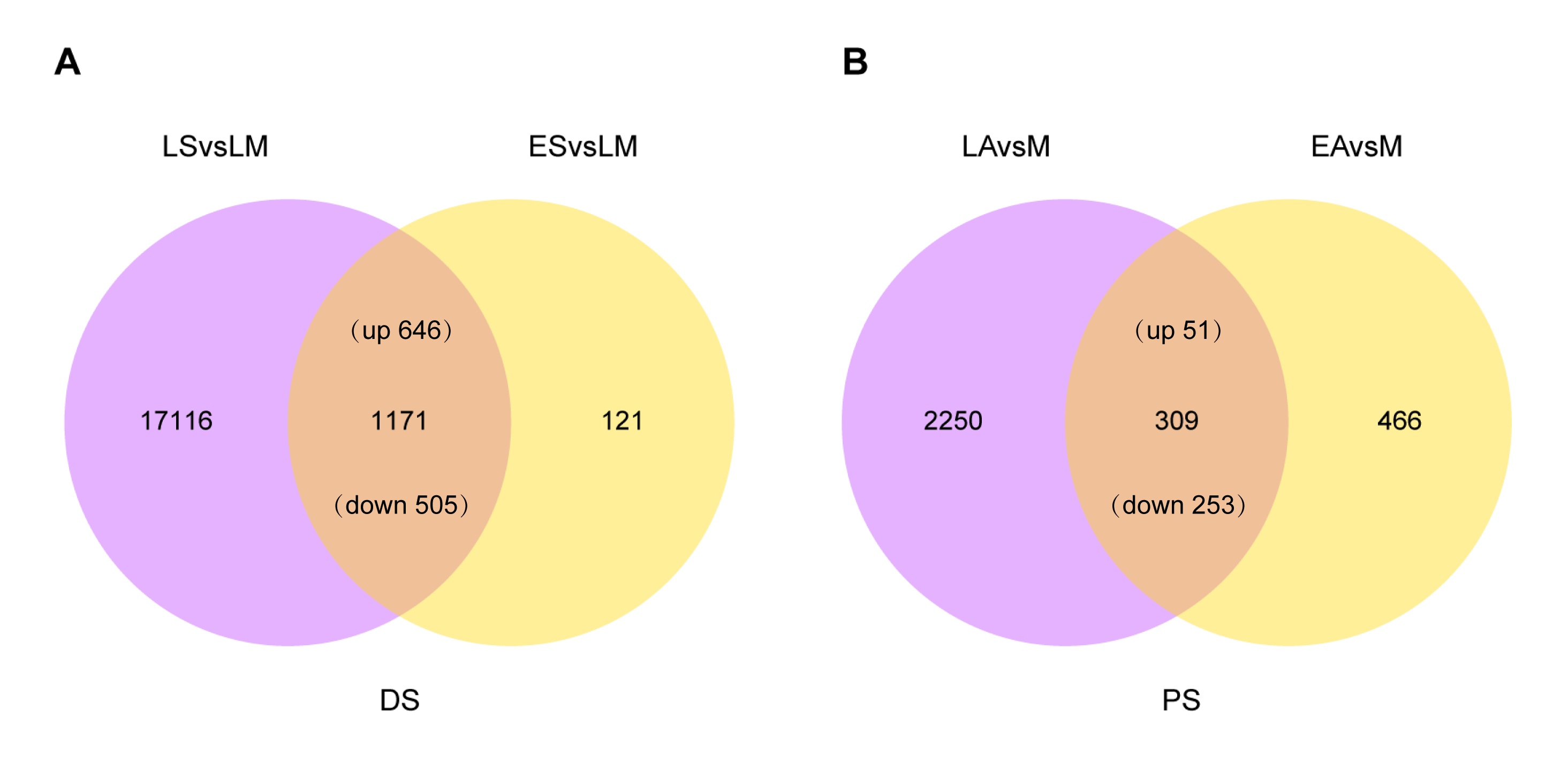

Supplement: Supplementary file 1 — Supplementary Figure S1. [file 41598_2020_77395_MOESM1_ESM.tif]

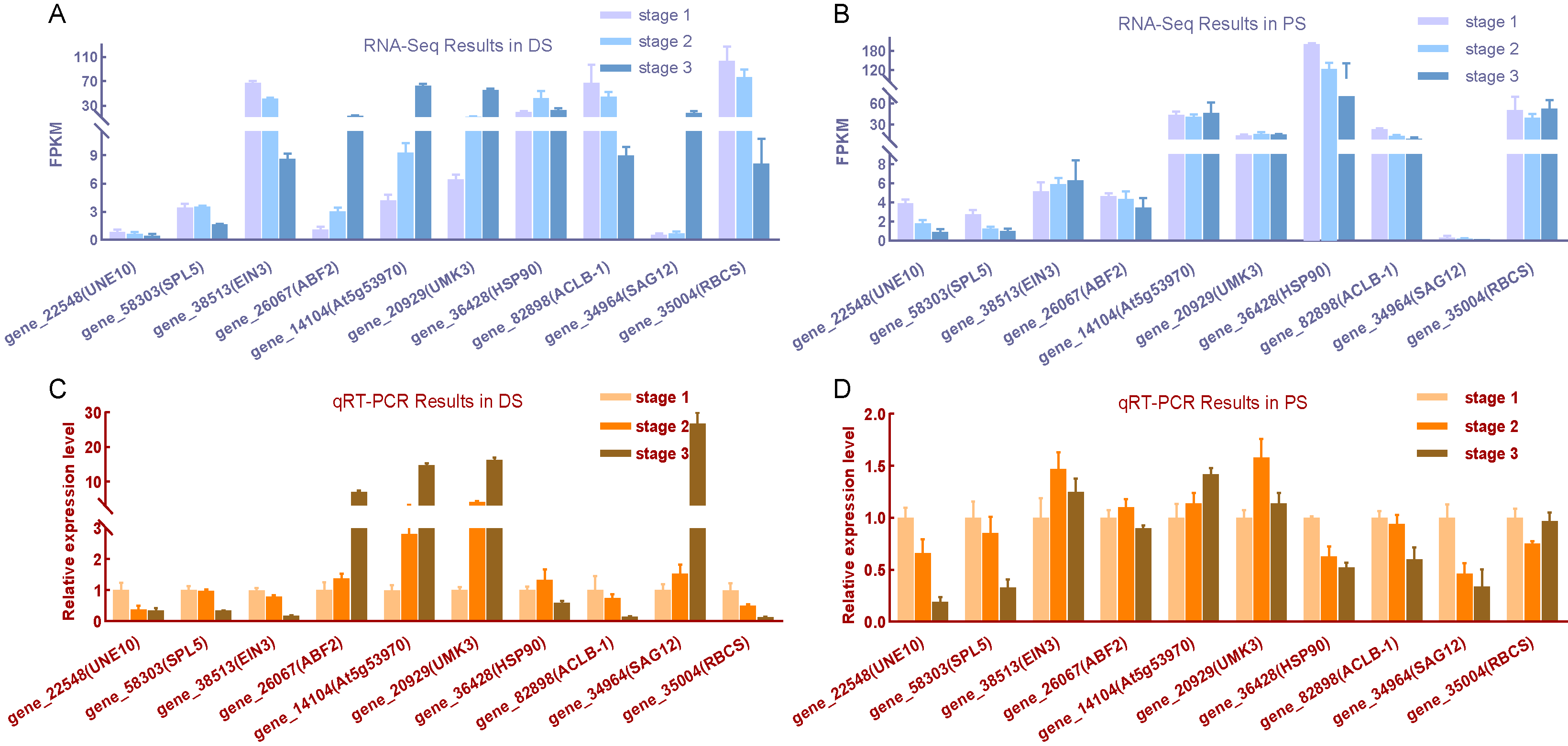

Supplement: Supplementary file 2 — Supplementary Figure S2. [file 41598_2020_77395_MOESM2_ESM.tif]
