# Supplementary material for: Transcriptome divergence between developmental senescence and premature senescence in Nicotiana tabacum L
Source: Sci Rep. 2020 Nov 25;10:20556. doi: 10.1038/s41598-020-77395-2 (PMC7688636; doi:10.1038/s41598-020-77395-2)
Supplement: Supplementary file 10 — Supplementary Captions. [file 41598_2020_77395_MOESM10_ESM.docx]

**Supplementary data**

Supplementary Figure S1 Veen of common DEGs.

Supplementary Figure S2 Results of RNA-Seq and qRT-PCR

Supplementary Table S1 Sumary of sequencing data.

Supplementary Table S2 Common DEGs in DS and PS respectively.

Supplementary Table S3 Results of KEGG enrichment.

Supplementary Table S4 List of transcription factors.

Supplementary Table S5 Results of qRT-PCR.

Supplementary Table S6 Meteorological data.

Supplementary Table S7 Primer list.
